# The Editor's Letter-Box

**Published:** 1913-03-29

**Authors:** Mabel C. Wells

**Affiliations:** 39 St. Stephen's Mansions, S.W.


March 18, 1913.

SIR,—Kindly omit the following words "and in some cases untrue" out of my letter which you received this morning; they are misleading.—Yours truly,

(Signed) MABEL C. WELLS.

39 St. Stephen's Mansions, S.W.,
March 18, 1913.

